# Proteomics Analysis Revealed that Crosstalk between *Helicobacter pylori* and *Streptococcus mitis* May Enhance Bacterial Survival and Reduces Carcinogenesis

**DOI:** 10.3389/fmicb.2016.01462

**Published:** 2016-09-15

**Authors:** Yalda Khosravi, Mun Fai Loke, Khean Lee Goh, Jamuna Vadivelu

**Affiliations:** ^1^Department of Medical Microbiology, Faculty of Medicine, University of MalayaKuala Lumpur, Malaysia; ^2^Department of Medicine, Faculty of Medicine, University of MalayaKuala Lumpur, Malaysia

**Keywords:** *Helicobacter pylori*, *Streptococcus mitis*, LC/Q-TOF mass spectrometry, phosphoglycerate kinase (PGK), thioredoxin (TrxA)

## Abstract

*Helicobacter pylori* is the dominant species of the human gastric microbiota and is present in the stomach of more than half of the human population worldwide. Colonization by *H. pylori* causes persistent inflammatory response and *H. pylori*-induced gastritis is the strongest singular risk factor for the development of gastric adenocarcinoma. However, only a small proportion of infected individuals develop malignancy. Besides *H. pylori*, other microbial species have also been shown to be related to gastritis. We previously reported that interspecies microbial interaction between *H. pylori* and *S. mitis* resulted in alteration of their metabolite profiles. In this study, we followed up by analyzing the changing protein profiles of *H. pylori* and *S. mitis* by LC/Q-TOF mass spectrometry to understand the different response of the two bacterial species in a multi-species micro-environment. Differentially-expressed proteins in mono- and co-cultures could be mapped into 18 biological pathways. The number of proteins involve in RNA degradation, nucleotide excision repair, mismatch repair, and lipopolysaccharide (LPS) biosynthesis were increased in co-cultured *H. pylori*. On the other hand, fewer proteins involve in citrate cycle, glycolysis/ gluconeogenesis, aminoacyl-tRNA biosynthesis, translation, metabolism, and cell signaling were detected in co-cultured *H. pylori*. This is consistent with our previous observation that in the presence of *S. mitis, H. pylori* was transformed to coccoid. Interestingly, phosphoglycerate kinase (PGK), a major enzyme used in glycolysis, was found in abundance in co-cultured *S. mitis* and this may have enhanced the survival of *S. mitis* in the multi-species microenvironment. On the other hand, thioredoxin (TrxA) and other redox-regulating enzymes of *H. pylori* were less abundant in co-culture possibly suggesting reduced oxidative stress. Oxidative stress plays an important role in tissue damage and carcinogenesis. Using the *in vitro* co-culture model, this study emphasized the possibility that pathogen-microbiota interaction may have a protective effect against *H. pylori*-associated carcinogenesis.

## Introduction

The human stomach was considered to be microbiologically sterile before the successful culturing of *Helicobacter pylori* from gastric biopsy tissue (Marshall and Warren, 1984). It was shown that gastritis and stomach ulcers in humans are caused by the Gram-negative, urease producing bacterium (Marshall and Warren, 1984). Later, it also became clear that this bacterium is a major risk factor in the development of gastric adenocarcinoma and mucosa-associated lymphoid tissue (MALT) lymphoma (Kusters et al., 2006). In developing countries, 70–90% of the population is infected with *H. pylori*; while in developed countries, the prevalence of *H. pylori* is 25–50% (Solnick et al., 2003; Obiageli and Ivan, 2016).

Besides *H. pylori*, the human stomach can also contain transient oral, esophageal or intestinal bacteria and is highly dominated by *Proteobacteria, Firmicutes, Actinobacteria*, and *Bacteroidetes* (Dicksved et al., 2007). These microorganisms may either be permanent members of the gastric microbiota but not picked up due to limitation of conventional microbiological culturing methods or may be in transit in the stomach (e.g., together with food intake). However, a change of the physiological conditions of the stomach, as occurs during acid-reducing drug therapy, corpus atrophy or gastric cancer, provides an opportunity for foreign microbes to enter and colonize the stomach (Dicksved et al., 2007).

Streptococci are members of the normal intestinal flora of healthy individuals which exert antagonistic activities against many intestinal pathogens (Heczko et al., 2006). A strong correlation was found between the presence of *Streptococcus salivarius* and *H. pylori* where 83% of the *S. salivarius* positive biopsies also harbored *H. pylori*. *S. salivarius* is known to have urease activity which creates a less acidic environment and could further enhance the survival and incidence of *H. pylori* (Ryan et al., 2008). Hence, streptococci may potentially survive and develop in an acidic gastric environment as an indigenous microbiota of the gastric mucosa, which may in turn inhibit the colonization by *H. pylori* (Adolfsson et al., 2004; Johnson-Henry et al., 2004; Uziel et al., 2004). In our previous studies, we have shown that *Streptococcus mitis* can be isolated from human gastric tissue biopsies (Khosravi et al., 2014a) and co-culturing *S. mitis* and *H. pylori* released metabolites that induced *H. pylori* to transform into viable but non-culturable (VBNC) coccoidal form *in vitro* (Khosravi et al., 2014b). On the other hand, culturability of *S. mitis* in the co-culture was enhanced. In this current paper, we completed our analysis by analyzing the changing protein profiles of *H. pylori* and *S. mitis* to understand the different response of the two bacterial species in a multi-species micro-environment. While it is not surprising that *H. pylori* changes to coccoid in a multi-species micro-environment, the enhancement of *S. mitis* survival capability deserve further investigation to determine any potential pathogenic role of this bacterium in the human gastric environment in the presence of *H. pylori*.

## Materials and methods

### Bacterial strains

*S. mitis* ATCC 6249 and *H. pylori* NCTC 11637 (ATCC 43504) obtained from the American Type Culture Collection (ATCC, USA) were selected as model microorganisms to simulate interaction in a multispecies micro-environment. Culturing of both organisms was performed on chocolate agar plates supplemented with 7% horse blood and was incubated at 37°C in a humidified incubator with 10% CO_2_ for 3 days (Khosravi et al., 2014b).

### Co-culture experiment

A bacterial co-culturing system was setup for this study in 12-well plates with a cell culture insert of 0.4 μm polyethylene terephathalate (PET) membrane (BD Biosciences, USA) that physically separate the two bacteria only allowing secreted compounds to penetrate as previously described (Khosravi et al., 2014b). Briefly, for the co-culture assay, 3 days old *H. pylori* and 1 day old *S. mitis* from chocolate-agar plates were used to make a suspension of OD600 ~0.02 (10^6^–10^7^ cfu/ml) and OD600 ~0.008 (10^5^–10^6^ cfu/ml) respectively in an enrichment medium of Brain heart infusion broth (BHI) supplemented with 0.4% yeast extract and 1% β-cyclodextrin. An aliquot of 2 ml suspension of *H. pylori* was distributed in each well of the 12-well plates. An aliquot of 0.5 ml suspension of *S. mitis* was added to the insert. The cultures were incubated at 37°C in a humidified incubator with 10% CO_2_ for 1–4 days. Experiments were carried out as independent biological triplicates.

### Protein extraction

The ProteoSpin detergent-free total protein isolation kit (Norgen Biotek, Canada) with the Halt protease and phosphatase inhibitors cocktail (Thermo Scientific, USA) was used for the isolation and purification of total protein from bacteria pellet according to the manufacturer's instructions. The lysates were subsequently treated with 10 mM dithiothreitol (DTT; Bio-Rad, USA) at 37°C for 10 min and alkylated with 55 mM iodoacetamide (IAA; Bio-Rad) for 30 min at room temperature. The proteins in the sample were digested with 1:50 (trypsin: protein) of MS-grade Pierce trypsin protease (Thermo Scientific, USA) at 37°C overnight. The samples were desalted using a Pierce C-18 spin column (Thermo Scientific, USA) and dried to completeness in a refrigerated CentriVap centrifugal vacuum concentrator (Labconco, USA) before mass spectrometry analysis.

### Protein profiling by LC/Q-TOF MS system

Tryptic peptides were analyzed on the 1260 Infinity HPLC-Chip System coupled with the 6540 UHD Accurate-Mass Quadrupole Time-of-Flight (Q-TOF) LC/MS systems (Agilent, USA). For analysis, the injection volume was 2 μl of tryptic digest (200 ng/μl). The HPLC-Chip was the Large Capacity C18 Chip (G4240-6210), which comprised a 160 nL enrichment column and a 150 mm analytical column. HPLC-grade water with 0.1% formic acid and acetonitrile with 0.1% formic acid were used as mobile phases A and B respectively. HPLC-grade acetonitrile and formic acid were procured from Friendemann Schmidt (Australia) and Sigma (USA), respectively. Instrument settings were as described in Chan et al. (2015).

### Data analysis

Mass spectrometric data were processed and analyzed using the Peaks software, version 7.5 (Bioinformatics Solutions Inc., Canada) for MS/MS-based identification and *de novo* sequencing. *De novo* sequencing was carried out with the default parameters, except that: (i) parent mass error tolerance was 1.5 Da, (ii) fragment mass error tolerance was 0.5 Da, (iii) trypsin as digestion enzyme, (iv) carbamidomethylation (+57.02 Da, C) as fixed modification, (v) oxidation (+15.99 Da, M) as variable modification, (vi) maximum variable post-translation modification allowed per peptide was three and (vii) *H. pylori* (strain NCTC 11637/ATCC 43504; 1633 proteins) and *S. mitis* (ATCC 6249; 1793 proteins) UniProtKB reference proteomes databases were used for identifications. Peptides were identified with PEAKS DB and filtered at 1% false discover rate (FDR). Proteins were filtered at 1 minimum unique peptide. Label-free quantification of protein abundances were estimated in each sample by correlation the average of the feature intensities of the three most highly responding peptides per protein.

### Statistical analysis

Statistical analyses were performed using the IBM SPSS version 21.0 software. One-way ANOVA and Two-tailed student's *t*-test were performed. *P*-value of <0.05 was considered significant.

## Results and discussion

**Figure 1:**
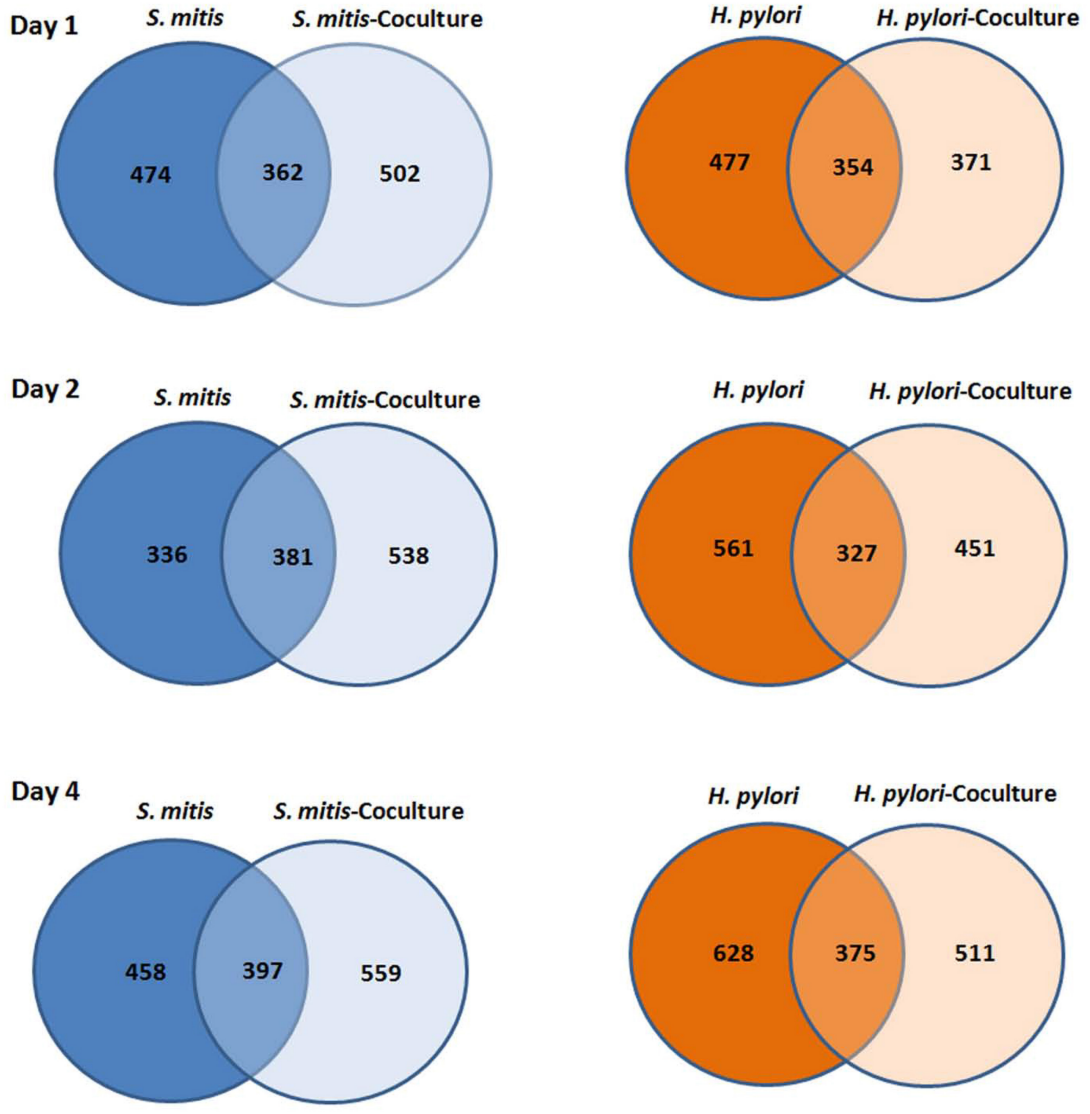
**Venn diagram of number of proteins ***H. pylori*** and ***S. mitis*** found in mono and co-cultures at day 1, 2, and 4**.

A total of 1514 *H. pylori* and 1414 *S. mitis* proteins - identified based on −logP ≥20, ≥1 unique peptide(s) (FDR <1%) and were detected in ≥2 of the triplicates - in both mono- and co-cultures (day 1, 2, and 4) are presented as Venn diagrams (Figure 1). Complete list of proteins identified can be found in the Supplementary Materials with confidence of identification and peptide data.

*H. pylori* proteins that were found to be significantly different between mono-cultured and co-cultured *H. pylori* were mapped to 12 and six biological pathways respectively (Table 1). Proteins involve in RNA degradation, nucleotide excision repair, mismatch repair, and lipopolysaccharide (LPS) biosynthesis were relative more abundant in co-cultured *H. pylori*. On the other hand, proteins involve in citrate cycle, glycolysis/ gluconeogenesis, aminoacyl-tRNA biosynthesis, translation, metabolism, and cell signaling were less abundant in co-cultured *H. pylori*. This is consistent with the observation that in the presence of *S. mitis, H. pylori* was transformed to coccoid (Khosravi et al., 2014b). *H. pylori* coccoid has been demonstrated to have low metabolic enzymes (FBA, EDD, AcnB, FumC, OorA, and ICD as shown in **Table 2B**) but proteins involved in DNA biosynthesis remained high (Loke et al., 2016). Despite that *H. pylori* coccoid cannot be cultured *in vitro*, it has been reported that the coccoid had a stronger inhibitory effect on proliferation and weaker apoptotic effect than its spiral counterpart (Li et al., 2013), which suggest that the coccoid may be an important factor in gastric cancer progression. However, contradictory to the earlier report (Loke et al., 2016), proteins involve in epithelial cell signaling during *H. pylori* infection were reduced and those involved in LPS biosynthesis (Table 1) were increased in *H. pylori* coccoid induced by co-culturing with *S. mitis*. These differences may highlight differences between *H. pylori* coccoids induced by various means (prolonged culturing vs. co-culturing with *S. mitis*) and age of coccoids (3 months old vs. 4 days old). Furthermore, the role of *H. pylori* coccoids in a multi-species environment and its impact on gastric pathogenesis has not been fully assessed.

**Table 1:** **KEGG pathway and GO enrichment analysis of ***H. pylori*** proteins in mono- and co-cultures using functional annotation tool of DAVID**.

**Sample group**	**Pathway**	**Count**	**Percentage**	**Proteins**	**Total**	**Pop Hits**	**Pop Total**	**Fold Enrichment**	***P*****-value**		
									**Bonferroni**	**Benjamini**	**FDR**
HP	hpp00020:Citrate cycle (TCA cycle)	9	6.02	AcnB, PorD, OorB, GltA, OorA, OorC, PorB, FumC, ICD	67	15	2109	16.79	2.22E−05	2.22E−05	1.41E−04
HP	hpj00970:Aminoacyl-tRNA biosynthesis	7	5.26	LeuS, ArgS, AspS, MetG, GatA, GLTX1, AlaS	67	25	2109	8.81	0.02	<0.01	0.10
HP	hpj00010:Glycolysis / Gluconeogenesis	6	4.51	PorD, FBA, PorB, GAP_2, ENO, JHP_1030	67	16	2109	11.80	0.02	<0.01	0.11
HP	hpj00230:Purine metabolism	6	3.76	JHP_1168, ADK, UreA, UreB, GuaB, NDK	67	34	2109	4.63	0.98	0.36	22.35
HP	hpj05120:Epithelial cell signaling in *Helicobacter pylori* infection	6	3.76	CagY, UreA, UreB, VacA, CagE, ORF15	67	34	2109	4.63	0.98	0.36	22.35
HP	hpp03010:Ribosome	5	3.76	RplI, RplN, RpsA, RplD, RplL	67	49	2109	3.21	0.10	0.67	56.63
HP	hpj00240:Pyrimidine metabolism	4	3.01	JHP_1168, NDK, TRXB_2, PyrF	67	35	2109	3.60	1	0.70	70.95
HP	hpj00030:Pentose phosphate pathway	3	2.26	FBA, EDD, TktA	67	13	2109	7.26	0.10	0.67	54.023
HP	hpj00250:Alanine, aspartate and glutamate metabolism	3	2.26	GlmS, AspB, AspA	67	13	2109	7.26	0.10	0.67	54.023
HP	hpj00480:Glutathione metabolism	3	2.26	PepA, GGT, ICD	67	6	2109	15.74	0.93	0.28	15.46
HP	hpj00620:Pyruvate metabolism	3	2.26	PorD, PpsA, PorB	67	12	2109	7.87	0.10	0.65	48.72
HPc	hpj00240:Pyrimidine metabolism	6	13.95	PyrG, DnaN, TRXB_1, DnaX, RpoB, PNP	24	35	2109	15.06	<0.01	<0.01	0.03
HPc	hpj00230:Purine metabolism	5	11.63	GppA, DnaN, DnaX, RpoB, PNP	24	34	2109	12.92	0.03	<0.01	0.41
HPc	hpj03018:RNA degradation	4	9.30	RNJ, PPK, RHO, PNP	24	10	2109	35.15	<0.01	<0.01	0.13
HPc	hpj03430:Mismatch repair	3	6.98	JHP_0847, DnaN, DnaX	24	15	2109	17.58	0.51	0.11	10.59
HPc	hpp00540:Lipopolysaccharide biosynthesis	3	6.98	KdsB, LpxA, LpxD	24	19	2109	13.88	0.67	0.15	16.32
HPc	hpj03420:Nucleotide excision repair	2	4.65	JHP_0847, UvrB	24	9	2109	19.53	0.10	0.55	63.38

*KEGG, Kyoto Encyclopedia of Genes and Genomes; HP, H. pylori mono-culture; HPc, H. pylori and S. mitis co-culture. Count, the number of genes associated with this gene set; percentage, calculated by “gene associated with this gene set “/” total number of query genes;” total, the number of genes in query list mapped to any gene set in this ontology; pop hits, the number of genes annotated to this gene set on the background list; pop total, the number of genes on the background list mapped to any gene set in this ontology; fold enrichment, the ratio of the proportions on query genes and the background information which are associated with the gene set; Bonferroni, Bonferroni adjusted p-value; Benjamini, Benjamini adjusted p-value; FDR, FDR adjusted p-value*.

Among proteins identified, 27 proteins satisfied the criteria to be selected for label-free quantification analysis using the Peaks software. In contrast to 23 proteins that were significantly different in expression level between mono- and co-cultured *H. pylori* (**Table 2B**), only 4 proteins were found to be significantly different between mono- and co-cultured *S. mitis* (Table 2A). This suggests that multi-species environment may have a greater impact on *H. pylori* than *S. mitis*.

**Table 2A:** **List of ***S. mitis*** proteins with significant expression difference in mono- and co-cultures**.

**Protein**	**KEGG Pathway**	**Unique peptide**	**Avg. Mass**	***S. mitis*** **monoculture**			***S. mitis*** **co-culture**			***P*****-value**		
				**Day 1**	**Day 2**	**Day 4**	**Day 1**	**Day 2**	**Day 4**	**Day 1**	**Day 2**	**Day 4**
50S ribosomal protein L13 (RplM)	smb03010:Ribosome	2	16143	0	0	0	0	0	6.82E+03	>0.05	>0.05	2.62E−08
UPF0297 protein RN80_02805	–	3	10227	5.15E+02	2.78E+02	0	1.73E+03	1.77E+03	8.30E+02	0.015	7.51E−04	>0.05
Phosphocarrier protein HPr (PtsH)	–	7	8939	3.50E+04	9.07E+03	1.25E+05	4.52E+03	3.76E+04	1.91E+03	>0.05	0.029	0.029
Phosphoglycerate kinase (Pgk)	smb00010:Glycolysis/Gluconeogenesis	3	41978	0	0	0	1.01E+03	8.57E+02	7.03E+01	0.028	>0.05	>0.05

**Figure 2:**
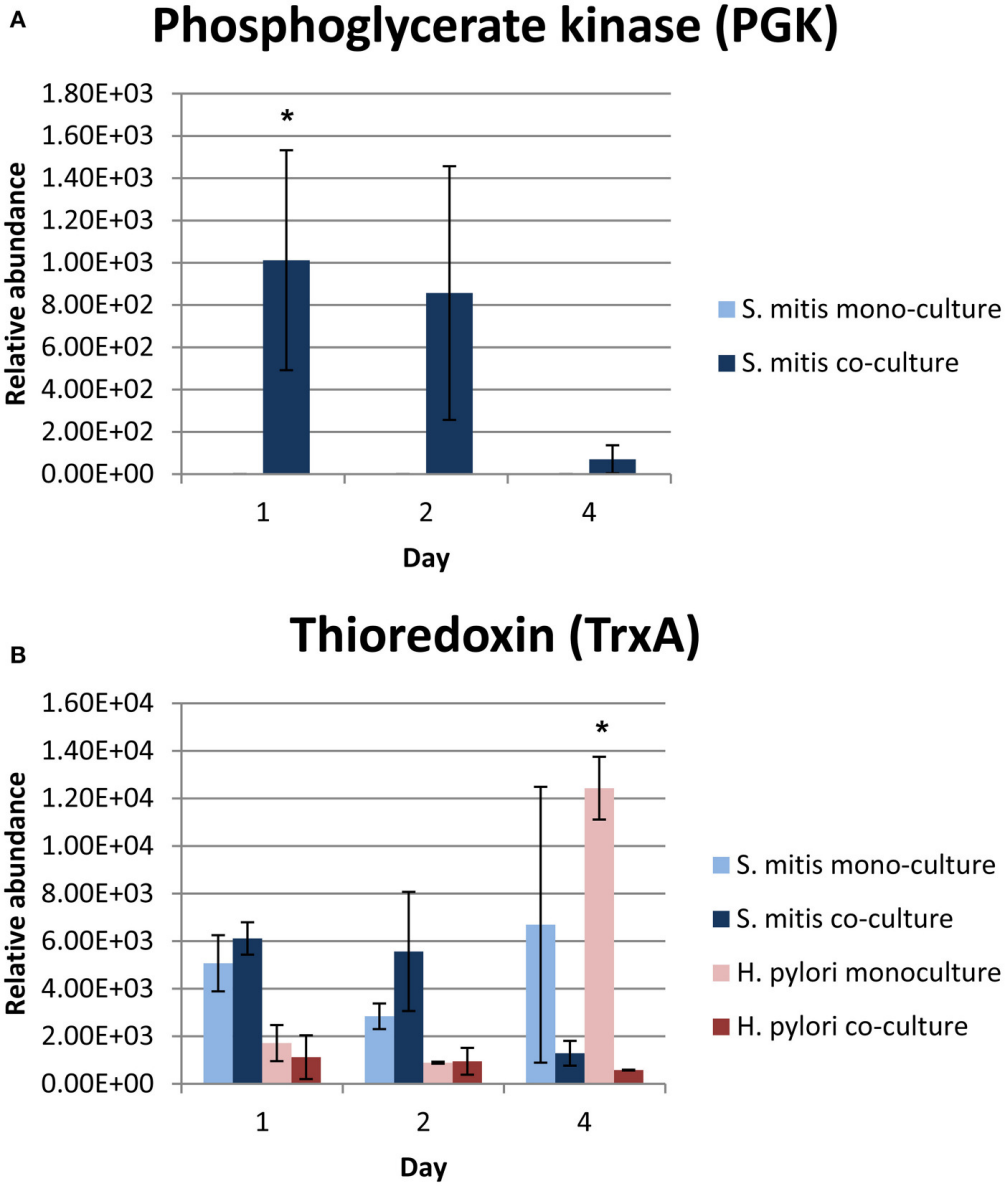
**Relative abundance of (A) PGK and (B) TrxA proteins in mono- and co-cultures**. ^*^Denote statistical significant differences with *p*-value = 0.028 (PGK) and <0.001 (TrxA) compared between mono- and co-cultures by 2-tailed one-way ANOVA.

Among the differentially expressed proteins, phosphoglycerate kinase (PGK), which is required for ATP generation in both the glycolytic pathway of aerobes and the fermentation process of anaerobes (Yoshida and Tani, 1983), was only expressed by co-cultured *S. mitis* (Table 2A and Figure 2A). In addition, PGK is one of the predominant surface-associated proteins of streptococci, such as *S. oralis* (Wilkins et al., 2003) and group B streptococci (Hughes et al., 2002). Interestingly, sera directed against PGK was shown to protect neonatal animals from *S. agalactiae* infection suggesting that this protein may be essential for multiplication or adhesion of streptococci *in vivo* (Hughes et al., 2002). The expression of *S. mitis* PGK in the presence of *H. pylori* might have contributed to the enhanced survival of *S. mitis*, which was demonstrated earlier by our group (Khosravi et al., 2014b).

Consistent with the reduction in abundance of enzymes involve in citrate cycle detected in the co-cultured *H. pylori* (Table 1), the expression of citrate cycle enzymes (AcnB, FumC, OorA, and ICD) were also found to be lower in co-cultured *H. pylori* (Table 2B), The citrate cycle is most sensitive to reactive oxygen species (ROS; Janero and Hreniuk, 1996). Thus, reduced level of expression of citrate cycle enzyme may indicate reduced oxidative stress response of *H. pylori* in the presence of *S. mitis*. This viewpoint is further supported by reduced expression of glutathione metabolism enzymes (ICD and PepA), thioredoxin (TrxA), flavodoxin (FldA), and thiol peroxidases (TPX and TsaA) in co-cultured *H. pylori*.

**Table 2B:** **List of ***H. pylori*** proteins with significant expression difference in mono- and co-cultures**.

**Protein**	**KEGG Pathway**	**Unique peptide**	**Avg. Mass**	***H. pylori*** **monoculture**			***H. pylori*** **co-culture**			***P*****-value**		
				**Day 1**	**Day 2**	**Day 4**	**Day 1**	**Day 2**	**Day 4**	**Day 1**	**Day 2**	**Day 4**
JHP_0156	–	2	27461	2.88E+02	6.69E+02	1.74E+02	1.21E+01	3.00E+01	3.67E+00	3.16E−06	8.92E−05	>0.05
Fructose-bisphosphate aldolase (FBA)	hpj00010:Glycolysis / Gluconeogenesis; hpj00030:Pentose phosphate pathway	12	33798	3.43E+02	6.43E+01	1.23E+04	9.77E+01	3.07E+00	4.73E+00	>0.05	>0.05	0.004
Phosphogluconate dehydratase (EDD)	hpj00030:Pentose phosphate pathway	4	66603	6.80E+02	8.46E+01	3.02E+03	1.93E+01	8.37E+00	0	6.19E−05	>0.05	0.008
Aconitate hydratase (AcnB)	hpj00020:Citrate cycle (TCA cycle)	11	92742	1.05E+03	2.02E+03	4.26E+03	5.37E+01	7.87E+01	0	0.046	8.61E−04	1.72E−04
Fumarase (FumC)	hpp00020:Citrate cycle (TCA cycle)	2	50920	0	0	1.98E+03	0	0	0	>0.05	>0.05	0.026
2-Oxoglutarate oxidoreductase subunit A (OorA)	hpp00020:Citrate cycle (TCA cycle)	4	41573	5.16E+02	5.96E+01	2.94E+03	0	0	0	>0.05	>0.05	0.025
Isocitrate dehydrogenase (ICD)	hpj00480:Glutathione metabolism; hpp00020:Citrate cycle (TCA cycle)	7	47462	6.92E+02	7.67E+02	1.76E+03	9.23E+01	3.06E+00	0.00E+00	>0.05	9.20E−04	>0.05
Aminopeptidase (PepA)	hpj00480:Glutathione metabolism	4	54612	1.40E+02	2.56E+01	1.63E+03	3.04E+01	2.71E+01	3.33E+01	>0.05	>0.05	0.002
Thiol peroxidase (TPX)	K11065 thiol peroxidase, atypical 2-Cys peroxiredoxin [EC:1.11.1.15]	6	18262	1.77E+03	5.40E+02	2.90E+03	1.26E+02	1.61E+02	0	>0.05	>0.05	0.003
Probable peroxiredoxin (TsaA)	K03386 peroxiredoxin (alkyl hydroperoxide reductase subunit C) [EC:1.11.1.15]; Exosome [BR:hpj04147]	5	22259	1.11E+03	6.15E+02	3.51E+03	0	0	0	0.007	>0.05	0.036
Thioredoxin (TrxA)	K03671 thioredoxin 1; Chaperones and folding catalysts [BR:hpj03110]	9	11855	1.71E+03	8.88E+02	1.24E+04	1.12E+03	9.47E+02	5.78E+02	>0.05	>0.05	9.98E−05
Flavodoxin (FldA)	K03839 flavodoxin I	9	17473	8.38E+03	4.88E+03	3.17E+04	3.25E+03	1.66E+03	6.83E+01	0.003	0.028	2.25E−05
JHP_0216	K03981 thiol:disulfide interchange protein DsbC [EC:5.3.4.1]; Chaperones and folding catalysts [BR:hpj03110]; Secretion system [BR:hpj02044]	3	29490	2.82E+02	2.11E+02	5.16E+03	6.35E+01	1.31E+02	4.10E+01	>0.05	>0.05	0.009
70kDa chaperone (DnaK)	hpj03018:RNA degradation	9	67122	3.03E+03	3.70E+03	4.75E+03	3.18E+02	0	0	2.15E−04	0.032	0.019
JHP_0301	K07226 heme iron utilization protein	3	28584	5.35E+01	2.01E+01	1.44E+03	4.83E+00	0	0	>0.05	>0.05	0.037
Response regulator (CheY)	hpj02020:Two-component system; hpj02030:Bacterial chemotaxis	5	13926	5.01E+02	1.09E+03	2.79E+03	0	0	0	>0.05	>0.05	0.020
Urease subunit B (UreB)	hpj05120:Epithelial cell signaling in *Helicobacter pylori* infection; hpj00230:Purine metabolism; hpj00220:Arginine biosynthesis	8	61684	4.21E+03	1.30E+03	7.23E+03	1.04E+02	0	0	6.49E−04	>0.05	2.39E−04
Urease subunit A (UreA)	hpj05120:Epithelial cell signaling in *Helicobacter pylori* infection; hpj00230:Purine metabolism; hpj00791:Atrazine degradation	7	26568	2.34E+03	4.23E+03	5.42E+03	1.79E+02	7.40E+01	7.83E+00	0.018	>0.05	0.025
50S Ribosomal protein L7/L12 (RplL)	hpp03010:Ribosome	2	13314	3.11E+03	1.19E+03	3.05E+03	0	0	0	1.90E−04	0.036	0.003
Elongation factor Ts (TSF)	Translation factors [BR:hpj03012]	2	39859	0	1.44E+02	3.55E+02	0	0	0	>0.05	>0.05	6.01E−04
Elongation factor G (FusA)	Translation factors [BR:hpj03012]	10	77127	3.02E+02	0	2.20E+03	3.07E+02	4.50E+01	9.61E+01	>0.05	0.027	0.002
Elongation factor Tu (TUF)	Translation factors [BR:hpj03012]; Exosome [BR:hpj04147]	10	43730	4.51E+03	1.19E+03	9.60E+03	0	8.83E+00	0	0.002	0.036	0.014
GTP-binding protein TypA/BipA homolog (TypA)	K06207 GTP-binding protein	3	66649	9.77E+01	2.93E+01	2.45E+03	0	6.23E+00	0	>0.05	0.026	0.023

The expression of thioredoxin (TrxA), a small redox-regulating protein that is involved in maintaining the thiol/ disulfide balance in both prokaryotes and eukaryotes (Holmgren, 1985), was significantly reduced in 4 days old co-cultured *H. pylori* (Figure 2B). This protein is essential for protecting bacteria, such as *Bacillus subtilis* (Scharf et al., 1998; Uziel et al., 2004), *Bacteroides fragilis* (Tally et al., 1975; Rolfe et al., 1997) and *Salmonella* species (Bjur et al., 2006), against oxidative stress for survival and replication. Interestingly, TrxA is also highly expressed in many cancers, including lung (Kim et al., 2003), cervix (Hedley et al., 2004), pancreatic (Han et al., 2002), colorectal (Raffel et al., 2003), hepatocellular carcinomas (Choi et al., 2002), gastric carcinomas (Grogan et al., 2000) and breast cancer (Cha et al., 2009). TrxA has been postulated to contribute toward cancer progression by playing crucial roles in maintaining cellular redox homeostasis and cell survival (Trachootham et al., 2008). Up-regulation of TrxA and related proteins has been postulated to present a dynamic redox change to drive proliferation and malignant progression of tumors (Karlenius and Tonissen, 2010). In the presence of *S. mitis*, the expression of *H. pylori* TrxA was reduced suggesting that *S. mitis* may potentially reduce the risk of *H. pylori*-associated gastric cancer development and/ or progression in the human stomach.

Alkylhydroperoxide reductase of *H. pylori*, which protects the bacterium from a hyperoxidative environment by reduction of toxic organic hydroperoxides, has been shown to function as a molecular chaperone for prevention of protein misfolding under oxidative stress (Chuang et al., 2006). This study highlights the importance of translation (elongation factors) and protein folding (chaperones) to *H. pylori* in response to oxidative stress. Thus, low level of expression of chaperones, such as TrxA, JHP_0216 and DnaK (aka 70 kDa chaperone; Table 2B), implies relatively low oxidative stress level in co-culture *H. pylori*. It has been shown that both bacterial factors and host inflammatory response causes oxidative stress on the gastric epithelium during *H. pylori* infection that may lead to apoptosis and tissue damage (Ding et al., 2007). Thus, low oxidative stress confers by *H. pylori* in a multi-species environment can be expected to be less pathogenic.

In conclusion, using *S. mitis* and *H. pylori* as model organism, data from this *in vitro* study suggest that in a multi-species setting, *S. mitis* may be able to benefit from cross-talking with *H. pylori* to enhancing its survival in the adverse gastric environment. Simultaneously, *S. mitis* may protect *H. pylori* from excessive oxidative stress. This *in vitro* co-culture model emphasizes the possibility that inter-species interaction may protect the host against bacterial-associated pathogenesis and carcinogenesis. However, this study is preliminary and the human gastric environment is highly complex and dynamic. Therefore, more evidences are required in order to fully understand the implication of *H. pylori*-gastric microbiota crosstalk and its impact on the development of gastroduodenal diseases in human.

## Author contributions

Conceived and designed the experiments: YK, MFL, KLG, JV. Performed the experiments: YK. Analyzed the data: YK, MFL.

## Funding

University of Malaya-Ministry of Higher Education (UM-MOHE) High Impact Research (HIR) grant (reference UM.C/625/1/HIR/MOHE/CHAN-02; account no. A000002-50001, “Molecular Genetics”).

### Conflict of interest statement

The authors declare that the research was conducted in the absence of any commercial or financial relationships that could be construed as a potential conflict of interest.
